# Real-Time Fluorescence-Based COVID-19 Diagnosis Using a Lightweight Deep Learning System

**DOI:** 10.3390/s26010339

**Published:** 2026-01-05

**Authors:** Hui-Jae Bae, Jongweon Kim, Daesik Jeong

**Affiliations:** 1 Department of Computer Science, Sangmyung University, Seoul 03016, Republic of Korea; baehj100@gmail.com; 2 Gyedang College of General Education, Sangmyung University, Seoul 03016, Republic of Korea

**Keywords:** fluorescence image-based, corona, deep learning, layer pruning, lightweight, edge device, NPU

## Abstract

The coronavirus is highly contagious, making rapid early diagnosis essential. Although deep learning-based diagnostic methods using CT or X-ray images have advanced significantly, they still face limitations in cost, processing time, and radiation exposure. In addition, for the possibility of real-time COVID-19 diagnosis, model lightweighting is required. This study proposes a lightweight deep learning model for COVID-19 diagnosis based on fluorescence images and demonstrates its applicability in embedded environments. To prevent data imbalance caused by noise and experimental variations, images were preprocessed using Gray Scale conversion, CLAHE, and Z-Score normalization to equalize brightness values. Among the tested architectures—VGG, ResNet, DenseNet, and EfficientNet—ResNet152 and VGG13 achieved the highest accuracies of 97.25% and 93.58%, respectively, and were selected for lightweighting. Layer-wise importance was calculated using an imprinting-based method, and less important layers were pruned. The pruned VGG13 maintained its accuracy while reducing model size by 18.9 MB and parameters by 4.2 M. ResNet152 (Prune 39) improved accuracy by 1% while reducing size by 161.5 MB and parameters by 40.22 M. The optimized model achieved 129.97 ms, corresponding to 7.69 frames per second (FPS) on an NPU(Furiosa AI Warboy), proving real-time COVID-19 diagnosis is feasible even on low-power edge devices.

## 1. Introduction

Coronaviruses (family Coronaviridae) are enveloped, positive-sense single-stranded RNA viruses that infect a wide range of hosts, including humans and animals. This viral family includes pathogens that have had a profound impact on human society in the past, such as severe acute respiratory syndrome (SARS) and Middle East respiratory syndrome (MERS). Among them, SARS-CoV-2 is the causative agent of coronavirus disease 2019 (COVID-19). COVID-19 is a highly contagious respiratory infectious disease, and clinical manifestations vary considerably among individuals. The most common symptoms include fever and cough, which may be accompanied by headache, myalgia, and generalized body pain. In some patients, the disease can progress to severe conditions such as dyspnea or chest pain and may even result in death. However, the majority of infected individuals experience mild to moderate symptoms and recover without requiring hospitalization [1].

Since its emergence, SARS-CoV-2 has continuously accumulated genetic mutations, and these changes have influenced viral transmissibility, infection patterns, and interactions with the host. The Alpha and Delta variants that emerged after the initial outbreak were reported to exhibit increased transmissibility and enhanced ACE2 binding affinity due to mutations in the spike protein. In contrast, the Omicron variant, which appeared later, has accumulated a substantially larger number of mutations and is known to possess a markedly enhanced immune evasion capability compared to previous variants [2]. This stepwise evolutionary process has further complicated the clinical characteristics of COVID-19 and has exposed the limitations of existing prevention and diagnostic strategies. During the early stages of the pandemic, SARS-CoV-2 was primarily understood to enter host cells through the angiotensin-converting enzyme 2 (ACE2) receptor. The Alpha and Delta variants were also reported to retain this ACE2-dependent entry mechanism while exhibiting increased infectivity and transmissibility. However, for more recent variants, including Omicron, a single entry pathway is insufficient to fully explain their infection characteristics. In particular, the Omicron variant has been reported to utilize multiple cellular entry pathways in addition to ACE2-mediated entry, including endosomal and lysosomal routes. This multivalent entry strategy is considered a major factor contributing to the expanded infection range and increased diversity of target tissues [3].

Such changes in cellular entry mechanisms and the accumulation of spike protein mutations are interpreted to be closely associated with a reduction in the infection-preventive effectiveness provided by existing vaccines and an increase in breakthrough infections. Indeed, several studies have reported that COVID-19 vaccines maintain an effectiveness of approximately 70% or higher in preventing severe disease even against variant viruses. However, for the Omicron variant and its subvariants, both infection-preventive efficacy and neutralizing antibody titers were shown to be markedly reduced compared to those observed for previous variants [2]. In particular, some Omicron subvariants have been reported to exhibit a reduction in neutralizing antibody responses to approximately half of the levels observed for earlier variants [2]. These findings suggest that while vaccines remain effective in reducing severe disease and mortality, they have limitations in completely preventing infection. Therefore, vaccination alone is insufficient to adequately suppress the spread of infection, and additional preventive strategies based on early diagnosis are required.

Because SARS-CoV-2 is highly transmissible, rapid and accurate diagnosis at an early stage is critical for suppressing the spread of infection. Currently, the most widely used diagnostic method is real-time reverse transcription polymerase chain reaction (RT-PCR), which detects SARS-CoV-2 by amplifying virus-specific genes [4]. RT-PCR provides high sensitivity and specificity and is used worldwide as the standard diagnostic test for COVID-19 infection [5]. However, this method requires expensive equipment and dedicated reagents, relies heavily on skilled personnel, and involves relatively long analysis times. To overcome these limitations, various molecular diagnostic methods, such as loop-mediated isothermal amplification (LAMP) [6] and CRISPR-based genetic diagnostic techniques [7], have been proposed. Nevertheless, these methods also have difficulty completely eliminating dependence on specialized equipment and reagents. Recently, to address these constraints, studies have been reported that utilize deep learning to classify pneumonia and COVID-19 based on chest CT images or chest X-ray images [8,9,10,11]. Although these approaches demonstrate high diagnostic accuracy, they have limitations in point-of-care diagnosis or real-time application due to issues such as the use of expensive imaging equipment, radiation exposure, and long processing times [12,13,14].

In addition, most complex deep learning models require a large number of parameters and high computational costs, making real-time inference difficult in lightweight devices or embedded environments [15]. Therefore, there is a need for lightweight model designs that minimize inference time and memory usage while maintaining accuracy. Accordingly, in this study, instead of CT- or X-ray image-based methods that require long acquisition times, a COVID-19 dataset based on fluorescence images acquired within approximately 5.5 min was utilized. These images were obtained using a plasma-based gold nanopillar multi-array gene amplification technique [16]. In such fluorescence-based PCR systems, the output signal is not a simple binary positive or negative result, but rather appears in the form of complex fluorescence images that reflect the dynamic patterns of the RNA amplification process.

These fluorescence patterns include spatial non-uniformity, weak signals at early stages, and subtle intensity variations, making reliable interpretation difficult through visual inspection alone. In particular, in point-of-care diagnostics or mass testing environments, the subjectivity and limited reproducibility of manual interpretation become more pronounced. Consequently, deep learning-based automated interpretation techniques are essential for objectively and consistently analyzing complex fluorescence patterns. In this study, data normalization techniques and lightweight deep learning models were designed, and the main contributions of this paper are as follows: (1) The effectiveness of normalization and contrast enhancement techniques for mitigating non-uniformity in fluorescence image data was analyzed. (2) A lightweight deep learning model incorporating model compression and quantization was designed and optimized for a neural processing unit (NPU) environment. (3) Inference speed and accuracy were compared across CPU, GPU, NVIDIA Jetson Nano, and NPU platforms to verify practical applicability in real embedded environments.

## 2. Related Works

### 2.1. Deep Learning Research on Various Image-Based COVID-19 Diagnostics

#### 2.1.1. Deep Learning Research on COVID-19 Diagnosis Based on CT Images

In the study by Shirin Kordnoori et al. [8], a deep learning framework was proposed for classifying COVID-19 infection using CT image data. The researchers combined seven publicly available datasets, including MedSeg, MosMedData, and COVIDx-CT, to construct the training dataset, and applied 90° and 270° rotation augmentation to address data imbalance. The proposed model was a convolutional neural network (CNN) consisting of ten convolutional layers and three fully connected layers. And batch normalization, the ReLU activation function, and dropout were used to prevent overfitting. The output layer performed binary classification to determine the presence of COVID-19 infection using the sigmoid function. Experimental results showed an accuracy of 89%, sensitivity of 95%, specificity of 88%, and AUC of 92%. Compared with models such as ResNet-50, DenseNet-169, and VGG-19, the proposed method demonstrated the best performance.

In the study by Honghua Liu et al. [9], a CNN–PSO hybrid model was proposed to classify COVID-19 based on CT image data. The researchers used public CT datasets such as the COVID-CT Dataset and the COVID-19 Radiography Database. To improve image quality, they applied normalization as well as data augmentation techniques including rotation and horizontal flipping. The proposed model consisted of two stages that combined a CNN-based feature extractor with particle swarm optimization (PSO). In the first stage, CNN extracted key features from lung CT images. In the second stage, the PSO algorithm was used to optimize the weights of the fully connected (FC) layer. Experimental results showed an accuracy of approximately 91.7%, an AUC of 92.8%, and a false positive rate (FPR) of 7.5%. Compared with a conventional CNN model, the proposed hybrid model achieved higher classification accuracy and a lower FPR. In particular, the PSO-based weight optimization process had a significant effect on reducing the FPR.

#### 2.1.2. Deep Learning Research on COVID-19 Diagnosis Based on X-Ray Images

In the study by Ens M.F. El Houby [10], a transfer learning-based deep learning framework was proposed to classify COVID-19 infection from chest X-ray images. The researchers used the publicly available COVID-19 Radiography Database as the dataset. To improve image quality, they applied several enhancement techniques, including histogram equalization (HE), contrast limited adaptive histogram equalization (CLAHE), and complementary color processing. They also utilized the lung masks provided in the database and trained the model with two types of input images: the original X-rays and the U-Net-based segmented images. VGG19 and EfficientNetB0 were used as base models. After freezing the pretrained weights through transfer learning, the fully connected (FC) layers were retrained. Experimental results showed that the VGG19 + CLAHE model achieved the best performance, with 95% accuracy, 96% sensitivity, 94% specificity, 94.12% precision, and an F1-score of 95.05. When segmented images were used, accuracy slightly decreased, which was interpreted as the result of losing additional information around the lung region that contributed to classification. In conclusion, this study demonstrated that even a relatively simple VGG19-based model combining image enhancement techniques and transfer learning can achieve high diagnostic accuracy for COVID-19 detection.

In the study by Qanita Bani Baker et al. [11], a deep learning framework was proposed to classify COVID-19 infection using chest X-ray images. The researchers combined two datasets—the Extensive and Augmented COVID-19 X-ray and CT Chest Images Dataset and a Kaggle dataset—to construct a balanced dataset of 15,000 images. They then applied 15 types of augmentation techniques, including zooming and rotation, to generate approximately 216,000 training images. Based on transfer learning, six CNN models were evaluated: Xception, Inception-V3, ResNet50, VGG19, DenseNet201, and InceptionResNet-V2. The experiments were conducted for both binary classification (normal vs. abnormal) and multi-class classification (normal, COVID-19, pneumonia). The Xception model achieved the best performance. For binary classification, it reached 98.13% accuracy, 98.14% precision, 97.65% recall, and an F1-score of 97.89%. For multi-class classification, it achieved 87.73% accuracy, 90.20% precision, 87.73% recall, and an F1-score of 87.49%. Furthermore, through the proposed augmentation and transfer learning strategy, other models such as ResNet50, VGG19, and DenseNet201 also outperformed their baseline versions.

#### 2.1.3. Deep Learning Research on COVID-19 Diagnosis Based on Fluorescence Images

In the study by Likun Zhang et al. [17], an AI-based CRISPR-Cas13a and total internal reflection fluorescence microscopy (TIRFM) system was developed to rapidly diagnose SARS-CoV-2 infection using fluorescence image data. Experimental data were obtained by capturing changes in fluorescence signals during the CRISPR-Cas13a reaction through TIRFM imaging. Three experimental conditions were used: a negative control, SARS-CoV-2 RNA at 100 pM, and SARS-CoV-2 RNA at 1 nM. For each condition, approximately 100 pairs of images were collected before virus injection (BeIn) and after injection (AfIn). To enhance the fluorescence difference between BeIn and AfIn images, several preprocessing methods were applied. The subtraction preprocessing (SP) method subtracted pixel values (BeIn − AfIn), while the division preprocessing (DP) method divided pixel values (BeIn ÷ AfIn). In addition, the setting and division preprocessing (SDP) method was introduced to improve DP by setting zero-value pixels in the AfIn image to one before performing the division. The study compared these three methods to determine which was more effective for SARS-CoV-2 detection. Four deep learning models (EfficientNet-B7, VGG-16, ResNet-152, and DenseNet-121) and three machine learning models (SVM, K-NN, and Decision Tree) were tested. Deep learning training was conducted in two stages using transfer learning. In the first stage, feature extraction layers were frozen and only the classifier was trained. In the second stage, all layers were fine-tuned. Each stage was trained for 20 epochs, with the initial learning rate set to 0.01 and reduced by 10% every seven epochs. As a result, the SDP method achieved the highest overall accuracy. Among the models, DenseNet-121, Decision Tree, EfficientNet-B7, ResNet-152, K-NN, SVM, and VGG-16 showed superior performance. These results confirmed that CNN-based deep learning models are effective for fluorescence image classification.

In the study by Shiaelis et al. [18], an AI diagnostic platform was proposed that combined single-particle fluorescence imaging with deep learning to identify multiple viruses, including SARS-CoV-2, within five minutes. In the experiment, viral particles were labeled by binding fluorescent DNA (Cy3, Atto 647N) through Ca^2+^ or Sr^2+^ ion mediation, and single-particle images were captured using TIRFM. The captured images were then analyzed by a 15-layer CNN that classified viruses based on fluorescence intensity distribution, morphological features, and axis ratio. The model was trained on various virus species, including SARS-CoV-2, Influenza A, Influenza B, IBV, and human seasonal coronaviruses (OC43, HKU1, NL63). Both infected samples and negative controls were included. The entire diagnostic process consisted of sample injection, fluorescence labeling, image acquisition, and deep learning-based classification, all completed within approximately five minutes. Experimental results showed classification accuracies ranging from 91% to 98%, successfully distinguishing SARS-CoV-2, Influenza A/B, and IBV. The system also achieved over 95% accuracy in distinguishing SARS-CoV-2 variants (Wuhan, Alpha, Delta). The authors emphasized that this platform enables near real-time detection without the need for RT-PCR, suggesting new possibilities for rapid, label-free molecular diagnostics. However, they noted limitations such as insufficient signal strength at low viral concentrations and the inability to distinguish symptom severity or infection stage.

Overall, previous CT and X-ray-based COVID-19 diagnostic studies achieved high accuracy but required expensive equipment, exposed patients to radiation, and involved long processing times, limiting their use for real-time or on-site diagnosis [10,11,12]. Therefore, in this study, we conducted deep learning research for COVID-19 diagnosis using a fluorescence reaction-based dataset.

### 2.2. Research on Deep Learning Model Lightweighting and Pruning Techniques

#### 2.2.1. Model Lightweighting Based on Layer Pruning

In the study by Sara Elkerdawy et al. [19], the researchers pointed out the limitations of the lightweight method using filter removal in terms of latency reduction and aimed to propose a lightweight method using layer removal. In a paper, they compared several methods for calculating layer importance to find the most optimal method. First, the statistical-based methods include weight statistics, Taylor weights, batch norm scale, and ensemble rank. Using these methods, the importance of each layer was calculated as the average value, and the layer with the lowest importance was removed. Second, the method proposed in this paper, the imprinting-based layer ranking method, calculates the layer importance based on accuracy. Lastly, they combined these two methods to remove layers with low importance.

Using these methods, they compared the VGG19-BN model on the ImageNet dataset and confirmed a 1.5% improvement. It was also confirmed that the imprinting-based layer ranking method consistently showed higher accuracy than other statistical criteria. In addition, this method showed higher performance in other models such as Shunet (7.3%), MobileNet (4.6%), MNASNet (2.8%), and ResNet18 (0.5%).

#### 2.2.2. Model Lightweighting Based on Quantization Techniques

In the study by Fatima Zahra Guerrouj et al. [20], the researchers proposed a quantization-based lightweight deep learning framework. The framework was designed to optimize object detection models on embedded GPU architecture for real-time applications such as autonomous driving. Based on YOLOv4, the model was quantized to FP32, FP16, and INT8 precision, and a real-time detection model was implemented on NVIDIA Jetson Nano and Jetson AGX Xavier. For this purpose, the KITTI dataset was used for training and evaluation. And the PTQ (Post-Training Quantization) method was applied to reduce model size, computational cost, and power consumption while maintaining detection performance. Model training was performed using the Darknet framework. After training, a three-stage conversion pipeline based on TensorRT was constructed, consisting of ONNX conversion, structure verification, and engine generation in FP16 and INT8 formats. At this stage, the model converted to INT8 reduced memory usage by more than 65%. It achieved a speed of 5 FPS on Jetson Nano and 62 FPS on Jetson AGX Xavier, showing performance close to real-time inference. In addition, the model size was reduced by 86.9% compared to FP32, proving the lightweight effect suitable for embedded systems. As a result, this study experimentally verified the improvement of computational efficiency and model compression through quantization techniques. It also presented a lightweight and high-efficiency model framework that enables real-time object detection even on embedded devices such as the Jetson series.

As such, the model lightweighting techniques based on layer pruning and quantization presented in previous studies play a key role in improving computational efficiency and real-time processing performance. Therefore, in this study, we apply these lightweighting techniques to a fluorescence reaction image-based deep learning model for COVID-19 diagnosis. The goal is to implement an efficient deep learning model capable of real-time inference even in lightweight embedded environments. The specific data acquisition and experimental configuration are described in detail in Section 3.

## 3. Methods

In Section 3, a fluorescence image-based diagnostic pipeline aimed at real-time COVID-19 diagnosis in low-power embedded environments is presented. The overall process consists of fluorescence dataset acquisition, data preprocessing and augmentation, deep learning model training, model lightweighting, and evaluation of inference performance in embedded environments.

### 3.1. Datasets

**Figure 1:**
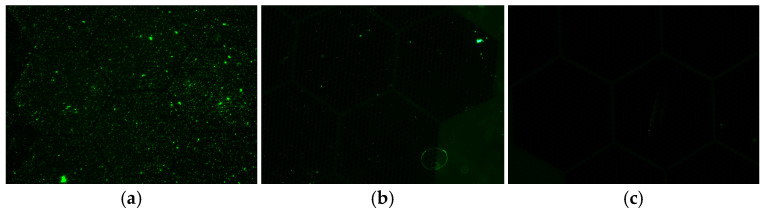
Coronavirus fluorescence expression datasets acquired using a plasma-based metal nanofiller device for ultra-fast gene amplification: (**a**) symptomatic individuals; (**b**) asymptomatic individuals; (**c**) healthy individuals.

In this study, the dataset was acquired based on the plasmon-driven gold nanopillar-based PCR platform proposed by Seo et al. [16]. This platform was designed to amplify and detect fluorescence signals generated during the gene amplification process in real time by utilizing the plasmon resonance effect on the surface of a gold nanopillar array. For dataset acquisition, SARS-CoV-2 RNA extracted from clinical specimens was injected into the platform together with the PCR reaction solution. After the amplification process, fluorescence signals generated by fluorescent dyes were captured using a camera mounted on a microscope, and fluorescence image data were obtained. Unlike conventional qPCR-based COVID-19 diagnostics, which require long processing times, this dataset was acquired using a photonic PCR platform that enables gene amplification and fluorescence detection within approximately 5.5 min. Therefore, it was selected as the dataset for this study because it is suitable for research on real-time COVID-19 diagnostic systems. As for the characteristics of the dataset, it consists of image data in which the presence of gene amplification is quantitatively represented at the molecular level through the distribution of fluorescence intensity. In a previous study [16], it was experimentally verified that the fluorescence intensity is proportional to the viral (RNA) concentration. The target virus in the dataset used in this study is coronavirus, and as shown in Figure 1, the dataset was categorized into three classes: symptomatic individuals, asymptomatic individuals, and normal (healthy) individuals.

### 3.2. Dataset Augmentation and Preprocessing

#### 3.2.1. Dataset Augmentation

The fluorescence-based COVID-19 dataset used in this study is based on SARS-CoV-2 RNA amplification reactions derived from actual patient samples. However, the process of directly acquiring infectious RNA and experimentally reproducing it requires ethical approval [16]. Due to these constraints, it is difficult to collect a large number of patient samples and to conduct repeated experiments. Therefore, in this study, data augmentation techniques were applied to secure diversity from a limited dataset. Fluorescence-based images can exhibit large variations in data distribution depending on experimental conditions, equipment characteristics, and noise patterns. Due to these characteristics, it is difficult to regard a specific augmentation combination as universally optimal under all conditions. Accordingly, a set of stable augmentation techniques that can be applied while preserving the structural and intensity patterns of fluorescence signal images was constructed. Because intensity-based patterns are the core features of fluorescence COVID-19 datasets, geometric transformations such as horizontal and vertical flipping and 90° rotation effectively expand data diversity without distorting the original signal. Random cropping reflects local signal non-uniformity, while Gaussian blur improves the robustness of the model against frequent defocus and optical blurring that occur during the experimental process. In addition, previous studies on fluorescence endoscopic image analysis have reported that geometric augmentations such as flipping and rotation have a significant effect on performance improvement, supporting the validity of the augmentation strategy adopted in this study [21].

**Figure 2:**
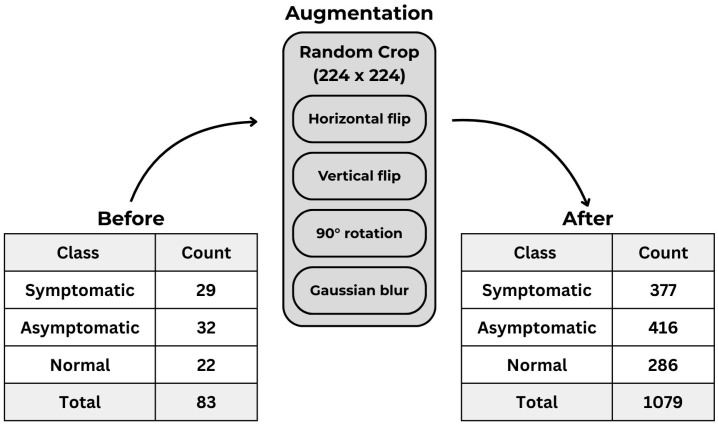
Data augmentation techniques used in this study and their results.

As shown in Figure 2, random cropping was applied to obtain images of size 244 × 244, and a total of 1079 images were generated using augmentation techniques including horizontal and vertical flipping, 90° rotation, and Gaussian blur.

#### 3.2.2. Data Preprocessing

**Figure 3:**
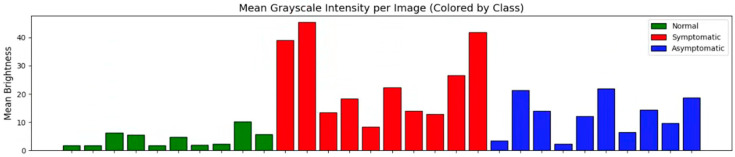
Nonuniformity in fluorescence intensity across classes, showing the mean grayscale brightness per image (10 randomly selected samples per class).

Although Seo et al. [16] effectively reduced thermal distribution non-uniformity through the design of the photonic PCR platform, it is difficult to completely eliminate subtle signal variations due to the inherent characteristics of fluorescence-based measurements. As a result of analyzing the actual dataset, variations in fluorescence intensity distribution were partially observed, as shown in Figure 3.

Therefore, in this study, various preprocessing methods were comparatively analyzed to correct such non-uniformity and to improve the generalization performance of the model so that it can operate stably under diverse input conditions in real-time diagnostic systems. First, grayscale conversion was applied based on the observation that pixel intensity is more critical than color information in fluorescence image-based datasets. By converting the three RGB channels into a single channel, the influence of unnecessary color variations was minimized. Second, contrast-limited adaptive histogram equalization (CLAHE) [22] enhances local contrast by dividing an image into small blocks and applying histogram equalization independently to each region, while preventing excessive noise amplification. Through this process, subtle differences in fluorescence intensity can be more clearly reflected. Finally, Z-score normalization [23] is a technique that normalizes data values (*x*) using the mean (*μ*) and standard deviation (*σ*). This method transforms the data to have a mean of 0 and a standard deviation of 1, thereby eliminating scale differences among variables.

(1) $$z=\frac{x-\mu }{\sigma }$$

**Figure 4:**
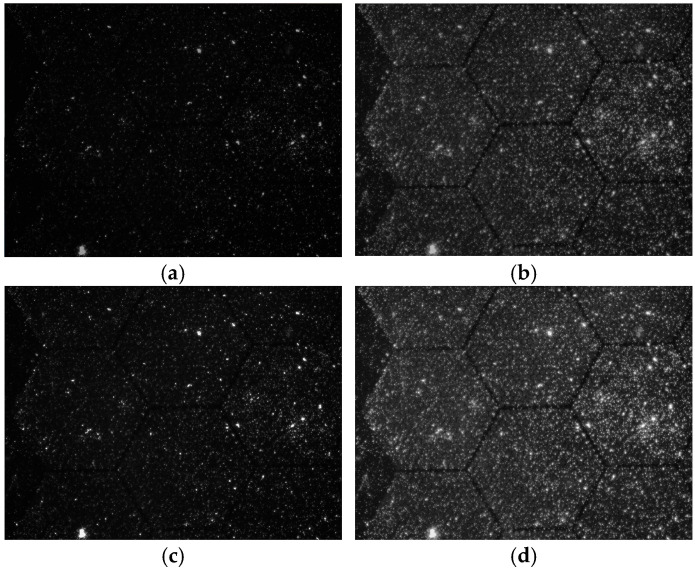
Comparison of dataset preprocessing methods based on symptomatic subjects (**a**) Gray Scale preprocessing; (**b**) Gray Scale + CLAHE preprocessing; (**c**) Gray Scale + Z-Score preprocessing; (**d**) Gray Scale + CLAHE + Z-Score preprocessing.

In this study, as illustrated in Figure 4, four preprocessing methods were compared and analyzed: grayscale, grayscale + CLAHE, grayscale + Z-score, and grayscale + CLAHE + Z-score. The analysis results confirmed that the combination of grayscale + CLAHE + Z-score provides both local contrast enhancement and normalization, making it the optimal preprocessing strategy. Accordingly, this combination was selected as the final preprocessing method for the experiments conducted in this study. Detailed results are described in Section 4.1, “Data Distribution and Performance Analysis by Preprocessing Methods”.

### 3.3. Base Models and Training Settings

#### 3.3.1. Base Models

Among various deep learning models, CNN-based architectures were selected for this study. The reason is that, unlike Transformer-based models, CNNs are designed to learn local spatial features from image or video data by stacking layers hierarchically [24]. This characteristic makes CNNs suitable for fluorescence image-based datasets, where signals occur in small regions and local variations are critical [25]. Previous fluorescence image-based COVID-19 diagnostic studies [17] also showed that CNN-based deep learning models achieved high classification performance. Therefore, in this study, four representative CNN models—VGG, ResNet, DenseNet, and EfficientNet—were used for training [26,27,28,29]. By comparing these different architectures, we aimed to evaluate the trade-off between model complexity and performance and verify their scalability for lightweight, real-time COVID-19 diagnosis.

The VGG [26] model, proposed by the Visual Geometry Group (VGG) at the University of Oxford, is a deep convolutional neural network. Its main feature is the repeated use of 3 × 3 convolution filters across multiple layers, forming a simple yet deep architecture. The ResNet [27] model, proposed by Kaiming He et al., introduced a residual learning framework. Each layer learns in the form of “input + residual,” allowing very deep networks to be trained stably. The DenseNet [28] model, proposed by Huang et al., connects each layer to all previous layers using dense connections. This structure promotes feature reuse, improves gradient flow, and reduces the number of parameters, enabling efficient operation. Finally, the Efficient [29] model, proposed by Mingxing Tan and Quoc V. Le, scales network depth, width, and input resolution in a balanced manner through compound scaling.

#### 3.3.2. Training Settings

In this study, transfer learning was performed using pre-trained models, in which only the classifier part was fine-tuned. First, experiments were conducted to identify which model family among VGG, ResNet, DenseNet, and EfficientNet is most effective for the dataset used in this study. For model training and validation, the dataset was divided into training, validation, and test sets at a ratio of 8:1:1, and identical training settings were applied to all models. All input images were resized to 224 × 224, and grayscale images were converted to three-channel RGB format for model input. During training, data augmentation techniques such as horizontal flipping, ±5° rotation, brightness and contrast adjustment, and Gaussian blur were applied. All images were normalized using a mean and standard deviation of [0.5, 0.5, 0.5]. The training configuration was unified with a batch size of 16, a learning rate of 1 × 10^−4^, and 20 epochs. The Adam optimizer and cross-entropy loss function were used for all experiments.

### 3.4. Layer Pruning-Based Model Lightweighting

Considering the characteristics of COVID-19 fluorescence PCR images, layer pruning is a lightweighting strategy that is more suitable for the data characteristics and on-device environment requirements of this study than fine-grained pruning at the channel or filter level. In fluorescence PCR images, low-level features such as intensity, spot-like patterns, and local distributions serve as key diagnostic cues, and these features are mainly extracted in the early layers. Therefore, a structural approach is required that preserves the initial feature extractors while reducing redundant representations in the middle and later layers. Layer pruning is effective in that it allows selective reduction in model depth while keeping the initial layers intact, thereby reflecting these data characteristics. In addition, this study targets environments that perform real-time inference on low-power edge devices such as Jetson Nano and NPU-based platforms. While fine-grained pruning at the filter or channel level may reduce the number of parameters, the computational structure often remains complex, resulting in limited actual speed improvement. In contrast, layer pruning directly reduces network depth, effectively shortening inference time. Furthermore, due to the relatively small size of clinically derived datasets, there are limitations in statistically stable evaluation of the importance of individual filters. By comparison, the imprinting-based layer pruning method proposed in [19] can evaluate the contribution of each layer to overall performance without iterative retraining, thereby improving the stability of structural selection. For these reasons, this study adopted the imprinting-based layer pruning method proposed in [19] to perform model lightweighting.

The imprinting method estimates the importance of each layer (or block) without training by approximating how much it contributes to the actual classification accuracy. Then, layers with smaller contributions are removed first. The formula for calculating the importance of each layer in the imprinting method is described as follows.

(1) Layer Output

Let the output feature map of each layer (*i*) be (*O_i_*). Since each layer has a different number of channels ($n_{i}$) and spatial size, adaptive average pooling is used to create a fixed embedding for comparison. As a result, the embedding (*E_i_*) has a size of (*d_i_* × *d_i_* × $n_{i}$) which is adjusted to match the total dimension (N).

(2) $$d_{i}=\mathrm{round}\left(\sqrt{\frac{N}{n_{i}}}\right)$$

(3) $$E_{i}=\mathrm{AdaptiveAvgPool}\left(O_{i},\;d_{i}\right)$$

(2) Class-wise Representative Vector Calculation

Given training dataset samples (*j =* 1, …, *D*), and class labels (*C_j_* ∈ {1, …, *C*}) the embeddings (*E_j_*) obtained from Equation (3) for each layer (*i*) are averaged by class to form class-wise representative vectors (*P_i_*).

(4) $$P_{i}[:,\;c]=\frac{1}{N_{c}}\sum_{j=1}^{D}I_{\left[c_{j}==c\right]}\;E_{j}$$

(3) Layer-wise Classification Accuracy Calculation

For each sample (*j*), the dot product between its embedding (*E_j_*) and each class representative vector is computed. The class with the highest score is taken as the prediction result. Using this process, the importance of each layer is determined.

(5) y^j=argmaxc∈ {1,…,C}  Pi[:,c]TEj

(4) Layer Importance Evaluation and Removal

After determining layer importance, layers whose importance is lower than or equal to that of the previous layer are removed.

In this study, based on the previously proposed optimal preprocessing method (grayscale + CLAHE + Z-score), the top two candidate models for lightweighting were selected according to F1-score performance among multiple trained models. For the selected models, layer importance was calculated and visualized, and model lightweighting was subsequently performed by applying layer pruning based on these results.

### 3.5. Quantization & Embedded Deployment

To verify the feasibility of real-time diagnosis in embedded environments, accuracy, model size, and processing speed were compared across various hardware platforms, including GPU, CPU, NVIDIA Jetson Nano [30], and NPU, as summarized in Table 1. The experimental hardware used in this study consisted of a GeForce GTX 1080 Ti as the GPU, an Intel^®^ Core™ i7-6700 as the CPU, and a FuriosaAI Warboy [31] as the NPU. A neural processing unit (NPU) is a device designed to reduce power consumption and to perform neural network operations at high speed [32]. To perform model inference on the NPU used in this study, a model quantization process was applied. Specifically, the PyTorch version 2.4.1 (CUDA 11.8)-trained models were converted to ONNX format with the opset version set to 13 or lower. Subsequently, mean squared error (MSE)-based asymmetric static quantization was applied, along with FP32-to-INT8 quantization and graph optimizations that fuse operations such as convolution and ReLU into a single kernel. Through these processes, efficient inference in the NPU environment was enabled. In addition, since the Jetson Nano is also a low-power edge device, TensorRT-based quantization was applied by referring to the method proposed in [20]. Specifically, the ONNX-converted models were transformed into FP32 and FP16 formats, respectively, and model size and inference time were compared to derive the optimal performance configuration for the Jetson environment. By comparing accuracy and processing speed not only on CPU and GPU platforms but also in embedded environments such as Jetson Nano and NPU, the efficiency of model lightweighting and the feasibility of real-time diagnosis were evaluated.

## 4. Results

The performance metrics used to evaluate the models were accuracy, precision, recall, and F1-score. Each evaluation metric is defined as follows [33].

(6) $$Accuracy\;A=\frac{TP+TN}{TP+TN+FP+FN}$$

(7) $$Precision\;P=\frac{TP}{TP+FP}$$

(8) $$Recall\;(sensitivity)\;R=\frac{TP}{TP+FN}$$

(9) $$F1-Score\;=2\;\frac{Precision\times Recall}{Precision+Recall}$$

Here, *TP* (true positive), *TN* (true negative), *FP* (false positive), and *FN* (false negative) represent classification outcomes based on the confusion matrix.

In addition, inference time refers to the time required for a model to receive input data and produce an output. Therefore, in this study, the batch size was set to 1, and the inference time for a single input image was measured to compare computational efficiency among models. Furthermore, the entire processing pipeline was divided into preprocessing, model inference, and postprocessing stages, and the time required for each stage was measured to compare model performance across different devices. In the preprocessing stage, the input data are normalized, transformed, and corrected before being fed into the deep learning model to make them suitable for processing. During the model inference stage, the trained deep learning model performs predictions based on the input data. Finally, in the postprocessing stage, the model outputs are converted into a format that can be interpreted by humans or utilized in real-world systems.

### 4.1. Data Distribution and Performance Analysis by Preprocessing Method

**Figure 5:**
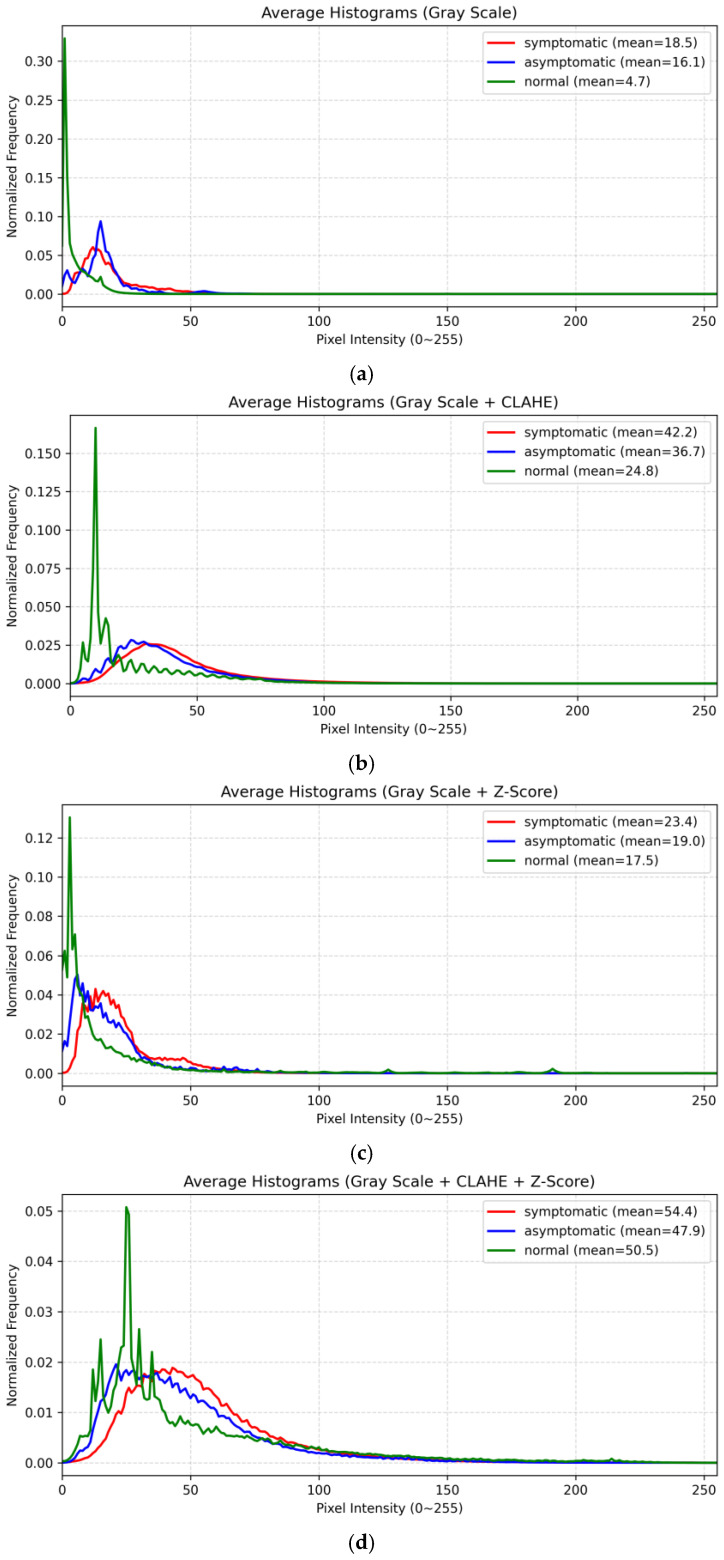
Comparison of class-specific dataset preprocessing methods using histograms: (**a**) Gray Scale preprocessing; (**b**) Gray Scale + CLAHE preprocessing; (**c**) Gray Scale + Z-Score preprocessing; (**d**) Gray Scale + CLAHE + Z-Score preprocessing.

Figure 5 shows a comparison of changes in the average histograms of three classes (symptomatic individuals, asymptomatic individuals, and normal individuals) according to different preprocessing combinations. In the basic grayscale method shown in Figure 5a, the distributions of all classes are concentrated in the low-intensity region (0–50), indicating that dataset non-uniformity remains largely uncorrected. In the grayscale + CLAHE combination shown in Figure 5b, excessive concentration in the low-intensity region is alleviated due to local contrast enhancement. However, because of the block-based processing characteristics of CLAHE, global alignment of brightness distributions is insufficient, and inter-image brightness variations still persist. In other words, this method has limitations in global scale correction.

In the grayscale + Z-score method shown in Figure 5c, global scale correction is achieved through normalization of the mean and variance. However, the limitation observed in the grayscale method, namely, the concentration of all class distributions in the low-intensity region (0–50) is not fully resolved. In contrast, the grayscale + CLAHE + Z-score combination shown in Figure 5d simultaneously applies local contrast enhancement (CLAHE) and global normalization (Z-score). This approach preserves subtle local intensity information while achieving global scale correction. As a result, the histogram exhibits the most stable and balanced distribution, indicating that dataset non-uniformity is effectively corrected. Based on this analysis, this combination was selected as the most optimal preprocessing method for model training in this study.

**Figure A1:**
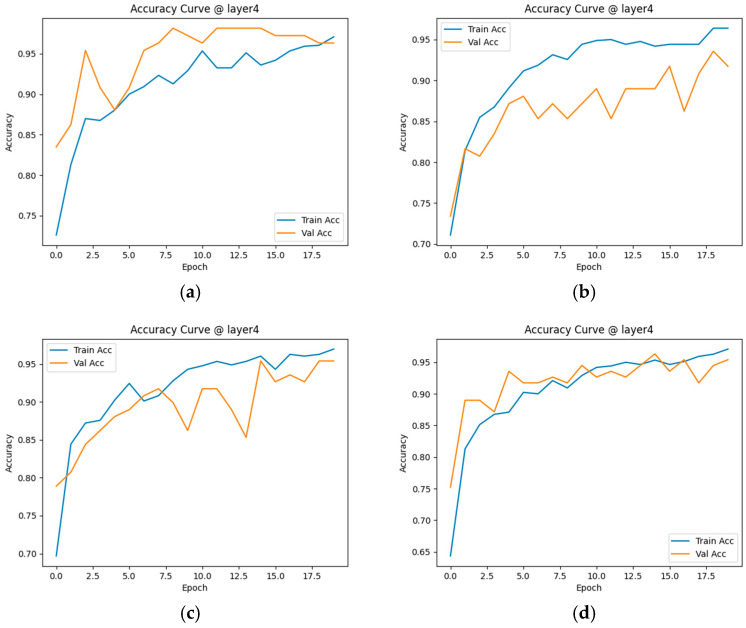
ResNet 101 acc graph by preprocessing method: (**a**) Gray Scale preprocessing; (**b**) Gray Scale + CLAHE preprocessing; (**c**) Gray Scale + Z-Score preprocessing; (**d**) Gray Scale + CLAHE + Z-Score preprocessing.

**Figure A2:**
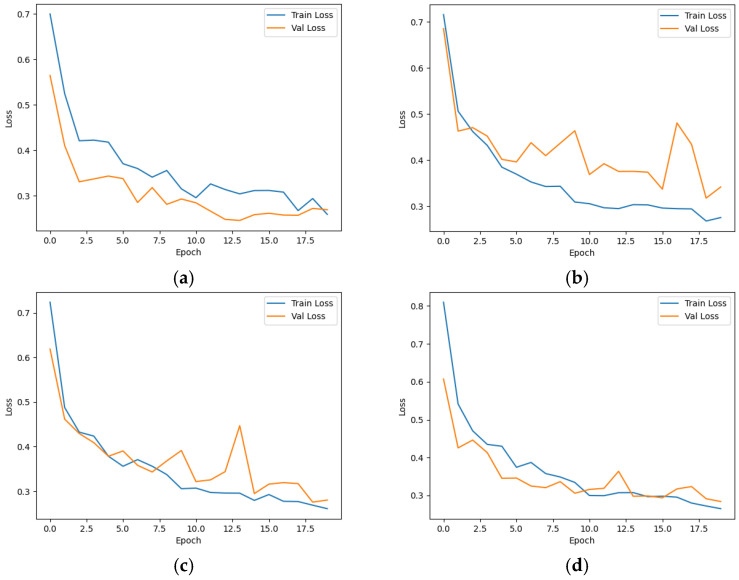
ResNet 101 loss graph by preprocessing method: (**a**) Gray Scale preprocessing; (**b**) Gray Scale + CLAHE preprocessing; (**c**) Gray Scale + Z-Score preprocessing; (**d**) Gray Scale + CLAHE + Z-Score preprocessing.

Additionally, as shown in Table 2, comparative experiments across models for each preprocessing method revealed that the simple grayscale approach achieved higher accuracy in some models. However, as illustrated in Figure A1 and Figure A2 of Appendix A.1, large fluctuations in validation accuracy and validation loss curves were observed, indicating a tendency toward overfitting [34]. The grayscale + CLAHE method was effective in enhancing local contrast through the application of CLAHE, but overfitting tendencies still remained. The grayscale + Z-score method improved training stability by normalizing brightness distributions, but unstable convergence was observed in certain regions.

In contrast, the grayscale + CLAHE + Z-score method simultaneously corrected both local and global non-uniformity in fluorescence intensity distributions, thereby improving data normality and training stability. Therefore, in this study, reproducibility and generalization performance were prioritized over absolute accuracy, and the grayscale + CLAHE + Z-score preprocessing method was ultimately selected for the experiments.

### 4.2. Performance Comparison and Analysis of Base Models

As shown in Table 3, under the preprocessing condition of grayscale + CLAHE + Z-score, model performance was observed to decrease in the order of the ResNet family, VGG family, DenseNet family, and EfficientNet family. In particular, within the ResNet family, ResNet-152 achieved an F1-score of 97.5%, while within the VGG family, VGG-13 showed the highest performance with an F1-score of 93.7%. The EfficientNet family exhibited relatively lower performance due to its lightweight model architecture, whereas the VGG and ResNet families demonstrated high performance owing to their deep feature extraction capability. Therefore, based on the F1-score under the grayscale + CLAHE + Z-score preprocessing condition, VGG-13 and ResNet-152, which showed the highest performance, were selected as target models for lightweighting.

Table 4 compares the parameter size, computational complexity (FLOPs), and model size of each model. In the EfficientNet family, the smallest model is only 16.4 MB, and most models maintain a lightweight structure of less than 100 MB. In contrast, the VGG family and ResNet family have relatively large model sizes ranging from 140 to 185 MB and from 44 to 233 MB, respectively. These results indicate that the EfficientNet family is designed to be highly computationally efficient. However, for the fluorescence-based COVID-19 dataset used in this study, lightweight models tended to suffer from performance degradation due to insufficient representational capacity. In other words, this dataset is better suited to deep CNN architectures that provide high-resolution feature representations. Considering that predictive performance for clinical applicability is prioritized over model compactness, ResNet-152 and VGG-13, which recorded the highest F1-scores in Table 3, were selected as models for lightweighting.

### 4.3. Comparison and Analysis of Layer Pruning-Based Lightweighting

#### 4.3.1. Imprinting-Based Layer Importance Analysis and Pruning Strategy

**Figure 6:**
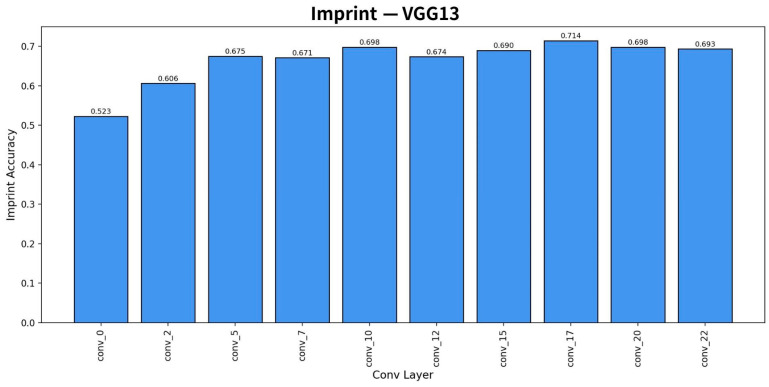
Visualization of importance by VGG 13 layer based on Gray + CLAHE + Zscore preprocessing.

For the VGG-13 model, lightweighting was performed based on layers with relatively low contribution identified through layer-wise importance visualization, as shown in Figure 6. In Figure 6, four Conv + ReLU operation blocks—Conv7, Conv12, Conv20, and Conv22—whose importance values were calculated to be lower than those of preceding layers were removed, and experiments were conducted accordingly.

**Figure 7:**
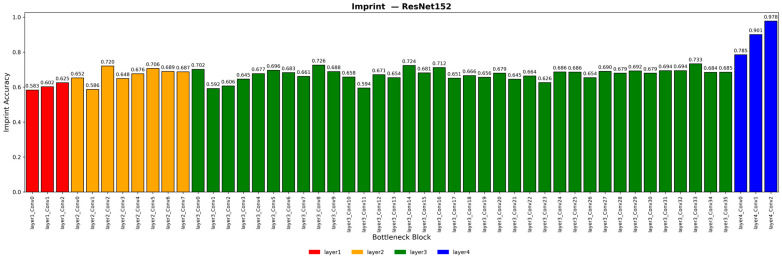
Visualization of Importance by ResNet 152 Layer Based on Gray + CLAHE + Zscore Preprocessing.

For the ResNet-152 model, three lightweighting strategies were explored based on the layer-wise importance visualization shown in Figure 7. The ResNet-152 model consists of four stages, each containing multiple bottleneck blocks. If only the four stages are visualized, the number of stages is too small to identify detailed layer importance. Therefore, the layer importance of ResNet-152 was visualized at the bottleneck block level, with the four stages distinguished by different colors. In this context, the terms “stage” and “layer” in Figure 7 are used interchangeably.

The first method applied the imprinting-based importance estimation proposed in [19] in which a total of 22 bottleneck blocks were removed. The second method was based on the observation that the difference between the maximum importance values of Layer2 and Layer3 was less than 0.03, which was interpreted as redundant learning. Accordingly, a total of 35 bottleneck blocks were removed, excluding the first bottleneck block of Layer3 (Layer3_Conv0). In the final method, the same 35 bottleneck blocks excluding Layer3_Conv0 were removed, and additional imprinting-based pruning was applied to other layers such as Layer1, Layer2, and Layer4, resulting in the removal of a total of 39 convolutional layers.

#### 4.3.2. Results of Layer Pruning-Based Lightweighting

As shown in Table 5 and Table 6, the lightweighting results indicate that, for the VGG-13 model, accuracy and recall were maintained, while precision and F1-score improved by approximately 1%. Similarly, for the ResNet-152 model, all performance metrics were either maintained or improved by approximately 1% compared to the baseline model across the various layer pruning strategies.

In terms of model size, number of parameters, and computational complexity, the layer-pruned VGG-13 model exhibited a reduction of approximately 20 MB in model size, a decrease of approximately 5 million parameters, and a reduction of approximately 1 GMac in computational cost compared to the original VGG-13 model. For the ResNet-152 model, applying layer pruning resulted in a maximum reduction of 161.5 MB in model size, a decrease of 40.22 million parameters, and a reduction of approximately 8.6 GMac in computational complexity.

**Figure A3:**
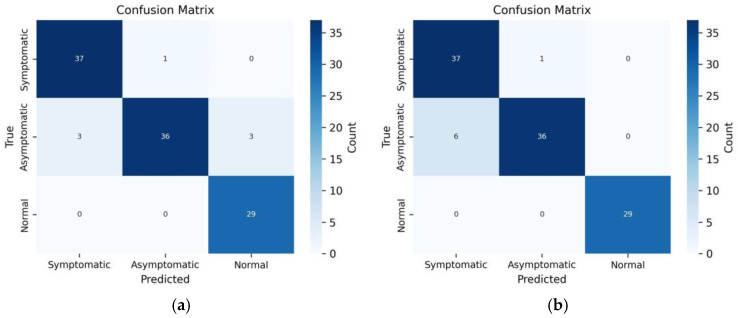

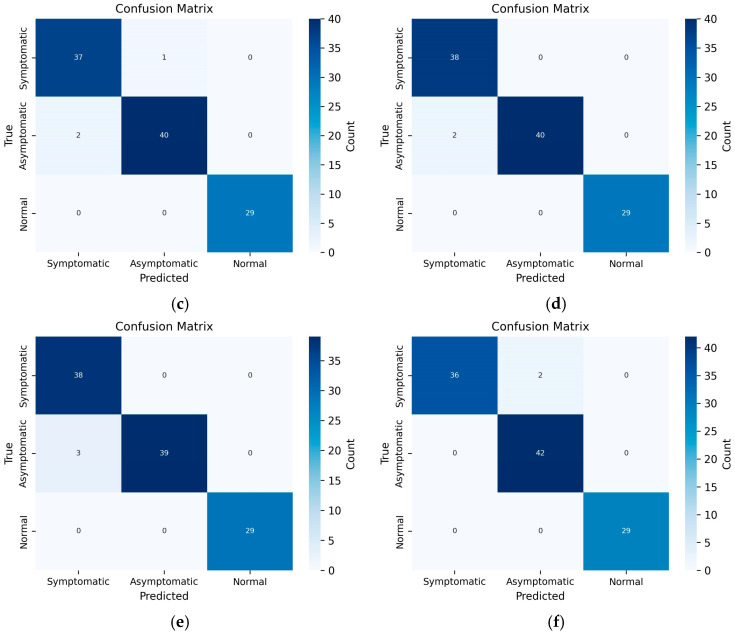
Confusion matrix of VGG13 and ResNet 152 lightweight models: (**a**) VGG13 model; (**b**) VGG13 (Prune Layer) model; (**c**) ResNet152 model; (**d**) ResNet152 (Prune 22) model; (**e**) ResNet152 (Prune 35) model; (**f**) ResNet152 (Prune 39) model.

These results demonstrate that the layer pruning technique effectively removes unnecessary computational blocks while preserving essential features. In addition, the confusion matrices corresponding to the results in Table 5 are presented in Figure A3 of Appendix A.2.

### 4.4. Device-Specific Inference Evaluation

#### 4.4.1. Analysis of Model Performance by Model Format and Device

To evaluate device-specific inference performance, model accuracy and model size were compared according to different devices and numerical precision formats, as presented in Table 7 and Table 8. As shown in Table 7, all models exhibited consistent classification accuracy on the GPU/CPU and Jetson Nano platforms regardless of whether FP32 or FP16 precision was used. In particular, both the original VGG-13 model and the layer-pruned VGG-13 model maintained identical accuracy across all formats and devices. For the ResNet-152 model, the layer-pruned variants achieved accuracy that was comparable to or slightly higher than that of the baseline model in the GPU/CPU and Jetson Nano environments. In contrast, when INT8 quantization was applied in the NPU environment, a slight degradation in accuracy was observed for the ResNet-152 models, whereas the VGG-13 model maintained the same performance. This result indicates that accuracy characteristics under INT8 precision may vary depending on the model architecture.

Table 8 presents the results of comparing model size across different devices and formats. When converting from the FP32 format to the FP16 format, the model size was reduced by approximately 50% across all models on both the GPU/CPU and Jetson Nano platforms. In addition, when the INT8 format was applied in the NPU environment, an additional model size reduction of approximately 50–70% was observed compared to the original FP32 models. Among the evaluated models, the ResNet-152 (Prune 39) model achieved the smallest model size while maintaining competitive accuracy, which shows that combining layer pruning with format conversion leads to a significant reduction in model size.

#### 4.4.2. Analysis of Model Inference by Model Format and Device

Table 9 presents a comparative analysis of inference latency for lightweighted models across different devices. For the VGG-13 model, the lightweighted version demonstrated the fastest inference speed in the order of GPU, NPU, Jetson Nano, and CPU. Conversely, the ResNet-152 model showed the highest inference performance in the order of NPU, CPU, GPU, and Jetson Nano. In particular, on the Jetson Nano, due to the characteristics of embedded environments, the FP16-based TensorRT format operated approximately 18 ms faster than the FP32 TensorRT format. In addition, on the Jetson Nano, the most lightweighted VGG-13 (Layer Prune) model and ResNet-152 (Prune 39) model achieved inference times of 15.3 ms and 37.0 ms, respectively, confirming the feasibility of real-time diagnosis on low-power hardware.

Table 10 compares the processing time of the ResNet-152 (Prune 39) model across various devices. The results demonstrate that real-time processing is achievable not only on GPU and CPU platforms but also on low-power edge devices such as the Jetson Nano and NPU. In particular, the NPU (INT8) exhibited the fastest overall processing time of approximately 129.97 ms, corresponding to 7.69 frames per second (FPS), indicating that the proposed model is suitable for real-time classification even in low-power edge device environments.

## 5. Discussion

This study proposed a lightweight deep learning-based diagnostic framework for analyzing fluorescence images generated from an ultrafast plasmon-driven gold nanopillar PCR platform. The experimental results confirmed that fluorescence images acquired within approximately 5.5 min contain sufficient diagnostic information for COVID-19 classification when appropriate preprocessing and model optimization are applied. Unlike conventional PCR methods that rely on endpoint fluorescence thresholding, the proposed approach exploits spatial fluorescence patterns reflecting the RNA amplification process, which are difficult to interpret using visual inspection or simple rule-based analysis. The results further indicate that preprocessing strategies play a critical role in stabilizing model training and improving generalization performance for fluorescence-based datasets. In particular, the combination of grayscale conversion, CLAHE, and Z-score normalization effectively mitigated both local and global non-uniformity in fluorescence intensity distributions. This is especially important for practical diagnostic systems, where experimental conditions, optical alignment, and noise characteristics may vary across measurements. By reducing sensitivity to such variations, the proposed preprocessing pipeline enhances the robustness of deep learning-based fluorescence image analysis in real-world settings.

From a model architecture perspective, the superior performance of deeper CNNs such as ResNet-152 and VGG-13 suggests that fluorescence reaction images require sufficiently expressive feature representations to capture subtle intensity distributions and localized signal patterns. While lightweight architectures such as EfficientNet offer computational advantages, their reduced representational capacity may limit performance in datasets where discriminative features are weak or spatially complex. The layer pruning results demonstrate that structural redundancy exists in deep models and can be effectively removed without degrading accuracy, or even with slight performance improvement, when layer importance is carefully evaluated. In addition, the real-time inference performance achieved on embedded platforms highlights the practical applicability of the proposed framework beyond laboratory environments. The successful deployment of pruned and quantized models on low-power devices such as the Jetson Nano and NPU suggests that fluorescence-based molecular diagnostics can be integrated with edge AI systems for point-of-care testing. Such capability is particularly relevant for large-scale screening scenarios, including emergency departments, mobile diagnostic units, and resource-limited environments, where rapid decision-making and system portability are essential.

Nevertheless, this study has certain limitations. The dataset used in this work was constrained in size due to the ethical and experimental challenges associated with collecting clinical RNA samples. Although data augmentation partially compensates for this limitation, it cannot fully represent the biological variability encountered in diverse clinical populations. Therefore, future work should focus on validating the proposed framework using larger and more heterogeneous datasets. Furthermore, while this study focused on COVID-19, the proposed pipeline is not disease-specific and may be extended to other fluorescence reaction-based molecular diagnostics, providing a foundation for more generalized real-time biosensing systems.

## 6. Conclusions

In this study, a lightweight deep learning model suitable for real-time diagnostic environments was proposed for fluorescence image-based COVID-19 data, with a focus on mitigating data non-uniformity. Due to the characteristics of fluorescence image datasets, imbalance in brightness and contrast may occur depending on experimental conditions such as photothermal uniformity and noise. To address this issue, various preprocessing methods were comparatively analyzed. As a result, the grayscale + CLAHE + Z-score combination simultaneously improved data normality and contrast, leading to enhanced training stability and generalization performance.

In terms of baseline model performance, the ResNet-152 base model achieved the highest performance, with an accuracy exceeding 97% and an F1-score of 97.5%. Among other models, the VGG-13 base model demonstrated the second-highest performance, with an accuracy exceeding 93% and an F1-score of 93.7%. Subsequently, convolutional layers were removed using an imprinting-based method and a strategy that removes layers with importance differences of less than 0.03. As a result, the ResNet-152 (Prune 39) model achieved an approximately 1% improvement in accuracy, while reducing model size by approximately 69% and FLOPs by approximately 74%. Similarly, the VGG-13 (Layer Prune) model maintained accuracy while achieving a reduction of approximately 13% in model size and approximately 8% in FLOPs.

Furthermore, to verify real-time diagnostic feasibility in embedded environments, GPU, CPU, NVIDIA Jetson Nano, and NPU platforms were compared. Based on optimal performance, inference speed followed the order of NPU, GPU, CPU, and Jetson Nano. In terms of overall processing speed, the ResNet-152 (Prune 39) model achieved the fastest performance on the NPU (INT8), with a total processing time of approximately 129.97 ms, corresponding to 7.69 FPS. These results demonstrate that the lightweight deep learning model proposed in this study achieves processing speeds suitable for real-time COVID-19 diagnosis even on low-power hardware. In future work, the proposed pipeline is expected to be extended to fluorescence reaction-based imaging data of other infectious diseases, enabling the development of a more generalized AI-based diagnostic system.

## Figures and Tables

**Table 1:** Model Deployment and Inference Environments for Different Hardware Platforms.

Hardware Platform	Device Used	Device-Specific Model Porting and Conversion Method
GPU	GeForce GTX 1080 Ti	PyTorch (FP32/FP16)
CPU	Intel^®^ Core^TM^ i7-6700	PyTorch (FP32/FP16)
Jetson Nano	NVIDIA Jetson Nano Developer Kit (4 GB) (Santa Clara, CA, USA)	PyTorch → ONNX → TensorRT (FP32/FP16)
NPU	Furiosa AI Warboy (Seoul, Republic of Korea)	PyTorch → ONNX → Furiosa NPU Compiler (INT8)

**Table 2:** Comparison of model accuracy by preprocessing method.

Model	Accuracy (%)			
	Gray Scale	Gray Scale + CLAHE	Gray Scale + Z-Score	Gray Scale + CLAHE + Z-Score
EfficientNet—B0	75.23	68.81	73.39	72.48
EfficientNet—B1	80.73	61.47	77.06	73.39
EfficientNet—B2	64.22	59.63	71.56	72.48
EfficientNet—B3	67.89	68.81	66.06	70.64
EfficientNet—B4	77.06	63.30	71.56	72.48
EfficientNet—B5	71.56	62.39	64.22	63.30
EfficientNet—B6	68.81	65.14	69.72	75.23
EfficientNet—B7	75.23	73.39	68.81	75.23
DenseNet 121	90.83	77.98	84.40	84.40
DenseNet 161	90.83	88.40	82.57	87.16
DenseNet 169	90.83	77.06	85.32	88.99
DenseNet 201	94.50	78.90	88.07	82.57
VGG 11	88.07	90.83	84.40	91.74
VGG 13	88.07	89.91	82.57	93.58
VGG 16	91.74	88.99	82.57	90.83
VGG 19	89.91	84.40	84.40	90.83
ResNet 18	94.50	94.50	94.50	94.50
ResNet 34	98.17	88.99	89.91	93.58
ResNet 50	99.08	93.58	97.25	92.66
ResNet 101	99.08	94.50	93.58	94.50
ResNet 152	99.08	92.66	98.17	97.25

**Table 3:** Performance comparison of deep learning models (based on Gray + CLAHE + Z-Score).

Model	Accuracy (%)	Precision (%)	Recall (%)	F1-Score (%)
EfficientNet—B0	72.48	73.60	73.30	73.44
EfficientNet—B1	73.39	74.31	74.31	73.97
EfficientNet—B2	72.48	74.16	72.97	73.34
EfficientNet—B3	70.64	70.05	70.90	70.26
EfficientNet—B4	72.48	74.30	72.24	72.99
EfficientNet—B5	63.30	65.54	64.53	63.27
EfficientNet—B6	75.23	75.23	77.02	75.66
EfficientNet—B7	75.23	75.69	76.48	75.85
DenseNet 121	84.40	84.71	85.76	85.02
DenseNet 161	87.16	87.91	88.39	88.12
DenseNet 169	88.99	89.34	90.14	89.62
DenseNet 201	82.57	85.34	84.75	82.97
VGG 11	91.74	91.65	92.33	91.91
VGG 13	93.58	93.47	94.36	93.70
VGG 16	90.83	91.05	91.81	91.30
VGG 19	90.83	91.42	91.10	91.25
ResNet 18	94.50	95.00	95.07	95.00
ResNet 34	93.58	94.23	94.28	94.17
ResNet 50	92.66	93.89	93.30	93.19
ResNet 101	94.50	95.16	94.53	94.70
ResNet 152	97.25	97.48	97.54	97.50

**Table 4:** Comparison of Model Size, Parameters, and Computational Complexity (based on Gray + CLAHE + Z-Score-based, pth–FP32).

Model	Model Size (MB)	Params (M)	FLOPs (GMac)	Efficiency (Acc/FLOPs)
EfficientNet—B0	16.4	4.01	0.41	176.78
EfficientNet—B1	26.5	6.52	0.60	122.32
EfficientNet—B2	31.3	7.71	0.69	105.04
EfficientNet—B3	43.4	10.70	1.01	69.94
EfficientNet—B4	71.0	17.55	1.56	46.46
EfficientNet—B5	114.4	28.35	2.44	25.94
EfficientNet—B6	164.3	40.74	3.47	21.68
EfficientNet—B7	256.9	63.79	5.52	13.63
DenseNet 121	30.6	7.48	2.90	29.10
DenseNet 161	111.7	27.60	7.85	11.10
DenseNet 169	54.4	13.34	3.44	25.87
DenseNet 201	77.7	19.08	4.39	18.81
VGG 11	141.8	35.44	7.55	12.15
VGG 13	142.5	35.62	11.26	8.31
VGG 16	163.7	40.93	15.43	5.89
VGG 19	185.0	46.24	19.59	4.64
ResNet 18	44.8	11.18	1.82	51.92
ResNet 34	85.3	21.29	3.68	25.43
ResNet 50	94.4	23.51	4.13	22.44
ResNet 101	170.7	42.51	7.86	12.02
ResNet 152	233.5	58.15	11.60	8.38

**Table 5:** Performance metrics of models after imprinting-based lightweighting.

Model	Accuracy (%)	Precision (%)	Recall (%)	F1-Score (%)
VGG 13	93.58	93.47	94.36	93.70
VGG 13 (Layer Prune)	93.58	94.45	94.36	94.17
ResNet 152	97.25	97.48	97.54	97.50
ResNet 152 (Prune 3)	98.17	98.33	98.33	98.33
ResNet 152 (Prune 35)	97.25	97.56	97.62	97.50
ResNet 152 (Prune 39)	98.17	98.48	98.25	98.32

**Table 6:** Comparison of model size and efficiency after imprinting-based lightweighting (pth FP32).

Model	Model Size (MB)	Params (M)	FLOPs (GMac)	Efficiency (Acc/FLOPs)
VGG 13	142.5	35.62	11.26	8.31
VGG 13 (Layer Prune)	123.6	30.90	10.33	9.06
ResNet 152	233.5	58.15	11.6	8.38
ResNet 152 (Prune 22)	144.9	36.08	6.77	14.50
ResNet 152 (Prune 35)	76.5	19.05	3.92	24.81
ResNet 152 (Prune 39)	72.0	17.93	3.03	32.40

**Table 7:** Comparison of model accuracy across different devices and formats after imprinting-based lightweighting.

Model	GPU/CPU		Jetson Nano		NPU
	pth (FP32) (%)	pth (FP16) (%)	engine (FP32) (%)	engine (FP16) (%)	enf (INT8) (%)
VGG 13	93.58	93.58	93.58	93.58	93.58
VGG 13 (Layer Prune)	93.58	93.58	93.58	93.58	93.58
ResNet 152	97.25	97.25	97.25	97.25	95.41
ResNet 152 (Prune 22)	98.17	98.17	98.17	98.17	95.41
ResNet 152 (Prune 35)	97.25	97.25	97.25	97.25	96.33
ResNet 152 (Prune 39)	98.17	98.17	98.17	98.17	96.33

**Table 8:** Comparison of model size across different devices and formats after imprinting-based lightweighting.

Model	GPU/CPU		Jetson Nano		NPU
	pth (FP32) (MB)	pth (FP16) (MB)	engine (FP32) (MB)	engine (FP16) (MB)	enf (INT8) (MB)
VGG 13	142.5	71.3	209.7	105.0	41.09
VGG 13 (Layer Prune)	123.6	61.8	157.3	78.8	34.95
ResNet 152	233.5	116.9	388.4	194.8	88.97
ResNet 152 (Prune 22)	144.9	72.5	217.0	108.9	66.22
ResNet 152 (Prune 35)	76.5	38.3	84.8	42.7	42.35
ResNet 152 (Prune 39)	72.0	36.0	76.0	38.3	39.72

**Table 9:** Comparison of Latency across different devices and formats after imprinting-based lightweighting (batch size = 1, averaged over 10 runs).

Model	GPU (ms)		CPU (ms)		Jetson Nano (ms)		NPU (ms)
	pth (FP16)	pth (FP32)	pth (FP16)	pth (FP32)	engine (FP16)	engine (FP32)	enf (INT8)
VGG 13	4.26	3.38	22.42	101.69	65.86	104.30	4.38
VGG 13 (Layer Prune)	3.40	2.73	20.49	90.62	65.24	81.85	4.25
ResNet 152	16.63	14.90	13.81	146.30	74.99	134.49	4.06
ResNet 152 (Prune 22)	9.93	8.75	8.38	90.79	47.04	84.84	3.01
ResNet 152 (Prune 35)	5.99	5.51	4.53	56.64	31.67	54.90	2.72
ResNet 152 (Prune 39)	4.83	4.24	3.54	48.66	27.00	45.24	2.47

**Table 10:** Comparison of Total Processing Time for the ResNet-152 (Prune 39) Model across Devices and Model Formats.

Processing Time (ms)—(Batch Size = 1, 10 Times Average)						FPS
Device	Format	Pre	Inference	Post	Total	
GPU	FP32	143.47	4.83	0.07	148.37	6.74
	FP16	143.47	4.24	0.07	147.78	6.77
CPU	FP32	143.84	3.54	0.04	147.42	6.78
	FP16	143.84	48.66	0.04	192.54	5.19
Jetson Nano	FP32	585.84	27.00	1.54	614.38	1.63
	FP16	585.84	45.24	1.54	632.62	1.58
NPU	INT8	127.41	2.47	0.09	129.97	7.69

## Data Availability

The data presented in this study are not publicly available due to institutional data-sharing restrictions.

## Abbreviations

The following abbreviations are used in this manuscript:

ACE2	Angiotensin-Converting Enzyme 2
Alpha	Alpha variant of SARS-CoV-2
Delta	Delta variant of SARS-CoV-2
LAMP	Loop-Mediated Isothermal Amplification
MERS	Middle East Respiratory Syndrome
Omicron	Omicron variant of SARS-CoV-2
SARS	Severe Acute Respiratory Syndrome
SARS-CoV-2	Severe Acute Respiratory Syndrome Coronavirus 2
COVID-19	Coronavirus Disease 2019
RT-PCR	Reverse Transcription Polymerase Chain Reaction
PCR	Polymerase Chain Reaction
qPCR	Quantitative Polymerase Chain Reaction
CRISPR	Clustered Regularly Interspaced Short Palindromic Repeats
CRISPR-Cas13a	CRISPR-associated protein 13a
CT	Computed Tomography
X-Ray	X-Radiation
CPU	Central Processing Unit
GPU	Graphics Processing Unit
NPU	Neural Processing Unit
Conv	*Convolution*
FC	Fully Connected
CNN	Convolutional Neural Network
VGG	Visual Geometry Group
DenseNet	Densely Connected Convolutional Network
ResNet	Residual Network
EfficientNet	Efficient Convolutional Neural Network
U-Net	U-shaped Convolutional Network
PSO	Particle Swarm Optimization
ACC	*Accuracy*
AUC	Area Under the Curve
FPR	*False Positive Rate*
HE	Histogram Equalization
CLAHE	Contrast Limited Adaptive Histogram Equalization
TIRFM	Total Internal Reflection Fluorescence Microscopy
RNA	Ribonucleic Acid
BeIn	Before In
AfIN	After In
SP	Subtraction Preprocessing
DP	Division Preprocessing
SDP	Setting and Division Preprocessing
SVM	Support Vector Machine
K-NN	K-Nearest Neighbors
AI	Artificial Intelligence
DNA	Deoxyribonucleic Acid
IBH	Infectious Bronchitis in Humans
BN	Batch Normalization
MNASNet	Mobile Neural Architecture Search Network
YOLOv4	You Only Look Once version 4
FP	False Positive
PTQ	Post-Training Quantization
ONNX	*Open Neural Network Exchange*
FPS	Frames Per Second
TP	True Positive
TN	True Negative
FN	False Negative
FLOPs	Floating Point Operations per Second

## Appendix A

### Appendix A.1

In Appendix A.1, to compare the stability of the training process, the changes in training and validation accuracy and loss are presented for each preprocessing method whose performance comparison results are shown in Table 2. Through this analysis, it was confirmed that some preprocessing methods exhibit tendencies toward overfitting and unstable convergence.

### Appendix A.2

In Appendix A.2, to supplement the classification performance results presented in Table 7, the classification performance of the models before and after lightweighting is presented in the form of confusion matrices. Through this analysis, prediction distributions and misclassification tendencies for each class were comparatively analyzed, and it was confirmed that classification balance is maintained even after model lightweighting.
